# Frontiers and Challenges in Electrochemical Corrosion Monitoring; Surface and Downhole Applications

**DOI:** 10.3390/s20226583

**Published:** 2020-11-18

**Authors:** Abuzar Khan, Ahsanulhaq Qurashi, Wael Badeghaish, Mohamed N. Noui-Mehidi, Md. Abdul Aziz

**Affiliations:** 1 Center of Research Excellence in Nanotechnology, King Fahd University of Petroleum and Minerals (KFUPM), Dhahran 31261, Saudi Arabia; abuzar@kfupm.edu.sa (A.K.); ahsanulhaq@kfupm.edu.sa (A.Q.); 2 Department of Chemistry, Khalifa University, Abu Dhabi 127788, UAE; 3 EXPEC Advanced Research Center, Saudi Aramco, Dhahran 31261, Saudi Arabia; wail.badegaish@aramco.com (W.B.); mohamed.nouimehidi@aramco.com (M.N.N.-M.)

**Keywords:** corrosion sensing and monitoring, sweet and sour corrosion, electrochemical methods

## Abstract

Corrosion sensing is essential to monitor and safeguard the materials’ health and control the maintenance cost of corrosion-prone materials used in various industries. The petroleum industry is a major sufferer of corrosion costs among various industries due to pipelines and downhole applications. This review article encompasses an overview of various technologies used in early detection stages for more reliable corrosion sensing and warnings. This review provides a summary of corrosion types, corrosion causing chemical species, different destructive and non-destructive technologies used in monitoring corrosion and a comprehensive overview of the state-of-the-art of various electrochemical techniques used for surface and downhole corrosion monitoring. Finally, the existing challenges for corrosion monitoring in surface and downhole conditions and prospects are discussed.

## 1. Introduction

Physical, chemical and/or electrochemical annihilation of a material by its atmosphere is termed corrosion [1]. Corrosion is initiated due to electrons’ movement from one material to another or a receiver material [2]. The corrosion of iron, de-alloying of aluminum, rusting of steels, pitting of alloys and stainless steels in salt containing media and so forth are all various forms of corrosion [2]. A new study entitled “International Measures of Prevention, Application and Economics of Corrosion Technology (IMPACT)” assessed the annual worldwide cost of corrosion at US $2.5 trillion [3]. This high cost indicates the importance of corrosion management. However, before corrosion management, there is an urge to develop reliable corrosion monitoring technology to search for better management solutions. Corrosion monitoring employs various techniques to conclude the corrosive atmosphere and at which rate metal loss is being observed. Corrosion measurement is the quantifiable technique by which the efficacy of corrosion control and inhibition methods can be assessed and offers the response to allow corrosion control and prevention approaches to be improved and calibrated [4]. Active corrosion management allows to act when required, while the severity of the problem is still small, thus helping to reduce the risk and cost [5]. In other words, monitoring is the first step towards identifying corrosion events and to devise targeted and optimized strategies to mitigate corrosion. Highly reliable corrosion sensing, together with chemical profiling under harsh conditions, remains an unresolved and grand challenge [5].

The qualitative and quantitative knowledge about on-going corrosion and chemical compounds present forms the basis of taking informed actions. This information is crucial to decide the type of solution needed. Conventional corrosion monitoring is an established technology and includes simple visual inspection, use of corrosion coupons, ultrasonic probes, electrochemical probes (i.e., electrical resistivity (ER) and linear polarization resistance (LPR)) and magnetic flux probes [6,7,8,9]. Currently, there are several options in the market that monitor corrosion in flow lines and pipelines. As an example, (i) corrosion coupon (ii) electrical resistance probe, (iii) electrochemical sensors and (iv) ultrasonic testing sensor are available from (i) Rohrback Cosasco Systems, (ii) Metal Samples Company, (iii) Rohrback Cosasco Systems/Corr Instruments, LLC and (iv) NDT Global LLC, respectively [10]. Information regarding the nature and amount of corrosives present continuously is either not or scantily available through fluid sampling and laboratory analysis [11,12].

To resolve these challenges of predominant corrosion sensor technology, there is irrepressible instinct to develop unique corrosion sensor with distinct essential properties such as simplicity of measurement and data interpretation, the precision of corrosion rate, applicability in a wide range of environmental corrosivity, response to a change in corrosion measurement, cost effective, reset mechanism, reliable, able to measure variable parameters. Since corrosion most often involves electrochemical processes, it is more reliable to focus on developing electrochemical sensors for different types of corrosion monitoring. In this review, our major focus is to present a broad prospective of various promising electrochemical methods employed for corrosion monitoring and the current progress and challenges related to corrosion sensing on the surface and downhole conditions, particularly employing electrochemical methods for corrosion monitoring applications

## 2. Type of Corrosion

There are various corrosion-related failures perceived in equipment and machinery used in oil and gas industry production and transportation facilities [13,14]. Crude oil contains numerous impurities, which are highly corrosive in nature. In oil and gas fields, the major sources of corrosion are carbon dioxide (CO_2_), hydrogen sulphide (H_2_S) and free water media [15]. The presence of these corrosive sources in fields makes internal constituents of equipment to suffer from corrosion. Particularly the inner lining and fittings undergo swift degradation. The materials finally suffer from loss of mechanical properties such as strength and ductility, which subsequently causes failure [13]. These losses eventually result in equipment breakdown or failure, which needs replacement and demands shutdown. Predominantly in petroleum industries, the foremost forms of corrosion comprise CO_2_ corrosion, H_2_S corrosion, O_2_ corrosion, galvanic corrosion, crevice corrosion, erosion corrosion, microbial corrosion and naphthenic acid corrosion [16,17].

### 2.1. CO_2_ Corrosion (Sweet Corrosion)

CO_2_ is one of the common corrosive agents in the oil and gas industry. CO_2_ corrosion starts by materials, medium and interphase related factors. Generally, carbon steel used in industries is less stable in water and CO_2_ and the rationale to use carbon steel is that the surface gets covered by a formation of a protective layer of oil, corrosion products, mineral scale or inhibitors if used or exists [18]. The corrosion process involves both cathodic and anodic reactions, but the existence of CO_2_ increases the corrosion rate by accelerating cathodic reaction kinetics. CO_2_ also forms carbonates that create thermodynamically stably iron carbonates. This can accelerate the anodic reaction of steel dissolution in slightly acidic electrolytes. Various mechanisms of CO_2_ corrosions have been proposed until now. However, each mechanism describes carbonic acid or bicarbonate ions’ formation due to the dissolution of CO_2_ in water containing media [19,20,21,22,23,24]. A number of models have been proposed to understand and predict CO_2_ corrosion [25]. In the cases of iron-based alloys, the reactions for the formation of CO_2_ corrosion in the absence of oxygen are given below in Equations (1)–(3) [9].

(1) $$\mathrm{Anodic}\;\mathrm{reaction}:\;\mathrm{Fe}\to {\mathrm{Fe}}^{2+}+2e^{-}$$

(2) $$\mathrm{Cathodic}\;\mathrm{reaction}:\;2{\mathrm{CO}}_{2}+2H_{2}O⇌2H_{2}{\mathrm{CO}}_{3}\underset{+2e^{-}}{\overset{2H^{+}2{\mathrm{HCO}}_{3}^{-}+2e^{-}}{\to }}H_{2}+2{\mathrm{HCO}}_{3}^{-}$$

(3) $$\mathrm{Total}\;\mathrm{reaction}:\;\mathrm{Fe}+2{\mathrm{CO}}_{2}+2H_{2}O\to \mathrm{Fe}+H_{2}{\mathrm{CO}}_{3}\to {\mathrm{Fe}}^{2+}+H_{2}+2{\mathrm{HCO}}_{3}^{-}$$

The profound impact of homogeneous reactions is primarily related to the CO_2_ hydration equilibrium, in which only a small proportion of CO_2_ (aq.) (∼0.2%) converts to H_2_CO_3_. Therefore, an extensive reservoir of CO_2_ (aq.) is present in the solution to restock the H_2_CO_3_ concentration as the corrosion process consumes it. The greater corrosion rates noted in aqueous CO_2_ solutions, in comparison to a robust acid solution (e.g., HCl) at the identical pH, were linked with the extra H_2_CO_3_ reduction and the impact of CO_2_ hydration reaction [26].

### 2.2. H_2_S Corrosion (Sour Corrosion)

H_2_S or sour corrosion is another major type of corrosion, which exists mainly in the oil and gas industry. H_2_S corrosion occurs when metal or carbon steel interacts with H_2_S comprising medium. Due to high pressure and temperature in oil wells, H_2_S dissolves in water and results in the formation of bisulfide (HS^−^) and sulfide (S^2−^) [27,28,29,30], which are very aggressive species for the corrosion of many metals. Sour corrosion is considered highly destructive in damaging the drill pipes. H_2_S becomes corrosive when it dissolves in water as a weak acid and produces hydrogen ions, which is corrosive in nature. Generally, iron sulphide and hydrogen—sour corrosion products result in pitting cracking [31]. Various mechanisms have been proposed for occurrence of sour corrosion. The general reaction for the formation of sour corrosion is shown in Equation (4) [32].

(4) $$H_{2}S+\mathrm{Fe}+H_{2}O\to \mathrm{FeSx}+2H+H_{2}O$$

### 2.3. Oxygen Related Corrosion

Oxygen reduction is one of the main corrosive components and is a common corrosion hazard faced by oil fields. Oxygen present in drilling fluids is the actual cause of corrosion of drilling equipment [33]. It is also corrosive in an aqueous atmosphere and being a powerful oxidant, reacts swiftly when encounters with metal. It acts as an electron acceptor in the cathodic process and enhances anodic degradation of metals even at a low concentration level of less than 55 ppm [34]. In the cases of iron-based alloys, the below reactions (Equation (5)) usually occur [13].

(5) $$2\mathrm{Fe}+2H_{2}O+O_{2}\to 2\mathrm{Fe}{\left(\mathrm{OH}\right)}_{2}$$

The generated Fe(OH)_2_ further oxidized with oxygen as described in Equation (6).

(6) $$4\mathrm{Fe}{\left(\mathrm{OH}\right)}_{2}+2H_{2}O+O_{2}\to 4\mathrm{Fe}{\left(\mathrm{OH}\right)}_{3}$$

This hydrous ferric oxide generates most of ordinary rust [13].

### 2.4. Galvanic Corrosion

Galvanic corrosion is a very simple form of corrosion and occurs when metals with different electrochemical potential come in contact with each other in the presence of a corrosive environment [35]. In this typical electrochemical reaction, metal with more negative potential acts as an anode and starts corroding [36] and the flow of electron equalizes when an anodic metal loses metal ions. There are various examples of galvanic corrosion in drilling processes, such as bronze and aluminum-based equipment used for oil production.

### 2.5. Crevice Corrosion

Crevice corrosion is a form of confined corrosion that occurs in gaps and narrow clearances due to immobile fluid/solution under microenvironment level [37]. The basic mechanism in this form of corrosion is the gradual acidification of solution in the interstices when exposed to the aerated chloride-rich solution. The O_2_ in the interstices is rapidly consumed due to restricted movement of a solution and gives rise to differential aerated cell between the de-aerated interstices and the aerated surface. As the dissolution of metal continues, more Cl^-^ ion migrates to compensate for excess metal ions and create a high Cl^-^ ion concentration. In crevice, acidification occurs, and the pH decreases and not increases. It may also occur that due to the saturation of ferrous and ferric ions in the crevice, the crevice becomes cathodic.

### 2.6. Erosion Corrosion

Erosion corrosion occurs due to the complete removal of the surface passivated layer. Usually, passive layers are ultrathin and are formed to protect corrosion and decrease the corrosion rate. However, due to equipment’s movement, stress and strain and huge turmoil, this passive layer detaches, enhancing corrosion rate significantly [38,39,40].

### 2.7. Microbial Corrosion

Microbial corrosion occurs due to the presence of microbes, such as bacteria in colonies [41]. These microbes produce waste products as CO_2_, H_2_S and organic acids, which gradually corrodes the equipment such as pipes. There are various reports which indicate the presence of microbes in the reservoir environment [42,43,44].

### 2.8. Naphthenic Acidic Corrosion

A non-aqueous process of corrosion occurred due to naphthenic acids is called naphthenic acidic corrosion (NAC). NAC is an issue for crudes refineries processing, which contain high concentrations of naphthenic acid. The vacuum and crude units of the refineries are mostly affected by NAC. The presence of sulfur compounds can change NAC’s mechanism, as demonstrated in the following reactions (Equations (7)–(9)) [45].

(7) $$\mathrm{Fe}+2\mathrm{RCOOH}\;\to \;\mathrm{Fe}{\left(\mathrm{RCOO}\right)}_{2}+{\;H}_{2}$$

(8) $$\mathrm{Fe}\;+\;R^{\prime \prime }\mathrm{SH}\;\to \;\mathrm{FeS}\;+{\;H}_{2}$$

(9) $$\mathrm{Fe}{\left(\mathrm{RCOO}\right)}_{2}\;+\;R^{\prime \prime }\mathrm{SH}\;\to \;\mathrm{FeS}\;+\;2{\mathrm{RCOOR}}^{\prime \prime }$$

where R” can be hydrogen or any organic chain.

## 3. Methods of Corrosion Detection

Various conventional indirect and direct methods have been employed for sensing and/or monitoring corrosion’s progress in well tubing/pipes, well casing strings and so forth [46,47,48]. These include visual methods, ultrasound/acoustic, radiographic, thermal imaging, electromagnetic, coupon, electrical resistance, electrochemical, electrochemical impedance spectroscopy (EIS) and chemical sensors [49,50,51,52,53,54,55,56,57]. Presently, the oil/gas industry monitors corrosion using conventional ER and ultrasonic probes for thickness monitoring. The working principle of ER is similar in function to electrical resistivity tomography (ERT) or electrical resistivity imaging (ERI). ER sensors use metallic electrodes in contact with the sample, both in solid or liquid phases and an alternating current is applied to the electrodes and the resulting alternating voltage is measured [58]. In ER probe, surface electrodes—made of the same material as the drilling or oil/gas transport pipe—are placed within boreholes or inside a vessel or pipe. Corrosion leads to the formation of a new phase and/or causes weight change, which changes the interface’s resistivity. As corrosion is the worsening of metal and oxide is forming on a corroded metal surface and increase the weight. But the oxides are not properly adherent to metallic surfaces and maybe, in the beginning, there is an increase in the weight but will finally lose metal and weight. Consequently, the corroded metal’s overall area will change. According to ohms law this will change the resistance of the metal under consideration. This change as a function of frequency (impedance) is continuously monitored. Any variance in the otherwise normal resistance, signals the onset of corrosion. On the other hand, ultrasonic probes monitor thickness. In general, these corrosion sensors suffer from various limitations such as slow response (months), less reliability, less accuracy of acquired data, expensive and difficulties in deployment to every locations, response limited to each chemical species/parameter and in particular ER and ultrasounds to have some disadvantages such as insensitivity towards minor changes and so forth. Among the above-stated corrosion monitoring methods, recently, electrochemical methods are more commonly used to monitor corrosion events. This review is mostly focused on the future prospects of various promising electrochemical corrosion detection methods and major challenges which encounter during these measurements.

## 4. Electrochemical Corrosion Monitoring of Various Substrates

Due to better selectivity, sensitivity, easy prototyping and low-cost, ease of fabrication, several types of electrochemical corrosion sensors have been developed. Therefore, widely used electrochemical techniques for corrosion sensing are LPR, Potentiodynamic Polarization (PDP), Open Circuit Potential (OCP), Electrochemical Noise (EN) technique, EIS, Harmonic Distortion Analysis (HDA), Potentiometric, Amperometry measurements and so forth. The corrosion sensor based on LPR are commercially available from Rohrback Cosasco Systems [10]. The principle of some of the methods is given below.

Prior to the electrochemical test performance using EIS, LPR, PDP and so forth, generally the working electrode’s potential is recorded with respect to a reference electrode without applying any external potential or current to the electrochemical cell. This recorded potential is called OCP. Once the system measures the cell’s OCP, it applies the desired working potential and the OCP that was already estimated to the working electrode. The OCP experiment specifies either a low or high corrosion probability but it does not provide the corrosion rate [59].

The scanning of the current potential (IV) domain to characterize a material medium pair can be carried out using the LPR method, which is a non-destructive technique [60]. In this method, minimal voltage variations (< ±30 mV to its corrosion potential) are applied to a metal. In this narrow potential range, the obtained current response is linear and therefore, the corresponding polarization resistance (R_p_), defined as the slope of the current vs. potential curve, is constant. R_p_ is inversely related to the instantaneous corrosion rate and can be described by the Stern-Geary equation [60,61], as described in Equation (10).

(10) $$R_{p}=B/i_{\mathrm{cor}}\;\mathrm{with}\;B=\left(\beta_{a}\beta_{c}/2.3\left(\beta_{a}+\beta_{c}\right)\right)$$

where, i_cor_ represents the corrosion current and β_a_ and β_c_ represent Tafel coefficients. If the constant B is known, then by using Faraday’s law, the corrosion rate can be determined from i_cor_.

Similarly, the PDP technique can also measure the corrosion rate by using the same LPR equation. Nevertheless, the PDP technique applies the metal wide voltage variations (> ±300 mV vs. OCP) and is considered a destructive process. PDP is perhaps the most widely used polarization testing technique to measure corrosion resistance. EN is a common term used to depict the current and potential fluctuations, which arise due to the electrode’s corrosion [62]. It is generated by electrochemical reactions, including the corrosion causing reactions. The researchers working in the corrosion field expressed the hope that the interpretation of EN would comprehend the corrosion process that cannot be achieved through other methods. Although this hope has not been fully implemented but there has been some progress and the technique has been in use for corrosion monitoring. EN analysis is a reliable technique that allows metal corrosion determination without any kind of external potential perturbation [63]. EN is more suited to detect and quantify localized corrosion [60].

On the other hand, an alternating voltage with varying frequencies (from MHz to μHz) is applied to the working electrode and the resulting current is measured in EIS measurements. Many impedances (Z) are recorded against each frequency range due to the phase differences of applied voltages and the resulting currents. The below equation describes the relationship between applied voltages, currents and phase angles shift, is shown in Equation (11).

(11) $$Z=\frac{E_{t}}{I_{t}}=\frac{E_{o}\sin\left(\omega t\right)}{I_{o}\sin\left(\omega t+\phi \right)}=Z_{o}\frac{\sin\left(\omega t\right)}{\sin\left(\omega t+\phi \right)}$$

where, E_t_, I_t_, E_o_, I_o_, ω and ϕ represent potential at time t, current at time t, signals amplitude, current at time zero, radial frequency and phase shift, respectively. These parameters can be determined by using the EIS equivalent circuit. Similarly, the ohmic resistance, double-layer capacitance (C_dl_) and charge transfer resistance (R_ct)_ can also be measured by using the EIS equivalent circuit. EIS provides the polarization resistance and to calculate the corrosion rate, the Stern Geary constant is necessary. However, the corrosion rate measurement by EIS is not so easy as LPR. Normally, the EIS method for corrosion monitoring is time-consuming due to the requirement of recording data at low frequencies [59]. Besides, the EIS data is not easy to evaluate and to calibrate [64]. This technique is also considered nondestructive [64].

Various types of electrochemical sensors have been developed to detect corrosion under different environments, such as corrosion sensors to monitor the corrosion of petroleum transfer pipeline (downhole and surface), brine, concrete and marine environment and so forth. Different chemical environment leads to different types of corrosion, as discussed above. For example, corrosion sensing in a petroleum transfer pipeline is quite difficult due to the highly corrosive environment. It carries hydrocarbon with more or less corrosive molecules like elemental Sulphur, H_2_S, CO_2_, H_2_O, inorganic salts and mass transfer with high velocity. Moreover, geometric downhole locations and elevated temperature are also important factors. Based on the corrosion environment, various electrochemical corrosion sensors have been developed and described below.

### 4.1. Corrosion Sensor for Monitoring Corrosion in Natural Gas Transmission Pipeline, Atmosphere, CO_2_ and Marine Environment

Covino et al. [65] reported electrochemistry-based corrosion sensors to study the internal corrosion of transmission pipelines for natural gas. The sensors probe in the form of flange were made using A106 steel pipeline and were tested in environments comparable to natural gas transmission pipelines. The sensors were sandwiched between the flanges on a 3-inch pipe and water and hydrocarbon volumes were allowed to pass through the pipe for a period of 24 h. SmartCET hardware was used for measuring the corrosion rate using the three different electrochemical techniques. The experimental data were collected, sorted and displayed by using a FieldCET software. The HDA, Tafel extrapolation and EN used to measure pitting factors, Stern-Geary constants and corrosion rates, respectively. The experimental results showed that the measurements were very sensitive to identify minor corrosion rates in the designated environments.

The field test at the test location was planned for almost 60 days of operation. During the overall experiments, it was determined that the electrochemical-based corrosion sensors could be utilized to work in a dry or wet gas environment, such as those encountered in the natural gas transmission pipeline. This approach is sensitive enough to detect and measure even small quantities of corrosion. These statistics can be summed up to give total weight loss of the metal and formulate corrosion problems in a well-developed manner.

Again Covino et al. [66] used flange and flush-mount probes to monitor internal corrosion in the natural gas transmission pipeline. They monitored the corrosion at three different sites under four different environments to mimic normal and upset conditions in a gas transmission pipeline. They showed that the LPR/HDA/EN techniques could measure the corrosion phenomenon in the natural gas transmission pipeline and associated environments to collect and transmit natural gas. HDA is a kind of transient analysis where single-frequency sinusoidal signals are applied to a source as input and the consequent distortion is recorded as output. It is used to detect general and localized corrosion processes [67].

**Figure 1:**
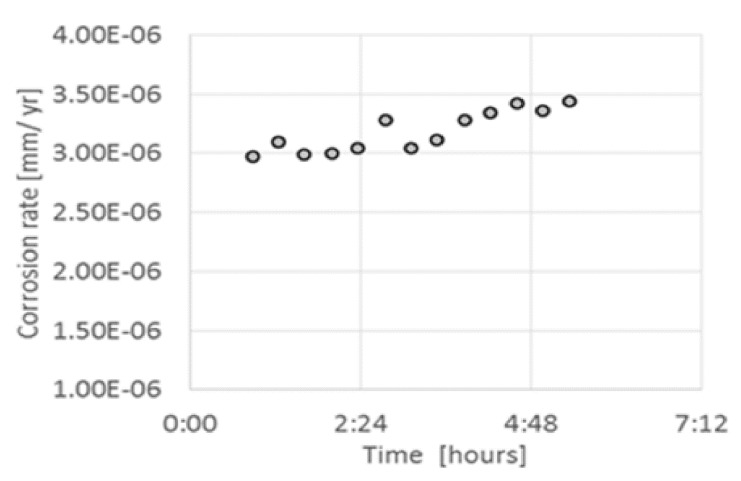
The corrosion rate of a carbon steel wire overtime via linear polarization resistance (LPR) on day 3 at 60 °C using the modified commercial cell. Reproduced with permission from ref. [68]. Copyright 2017 IFSA Publishing.

In another study for measuring the corrosion rate in natural gas pipeline, Beck et al. [68] designed a solid-state sensor using an ion conducting membrane to measure corrosion and conductivity up to the pressure of 1000 psi. The corrosion measurements were conducted using a carbon steel electrode and commercial Nafion^®^ membrane in the industrial conductivity cell at 0.69 MPa in nitrogen for 60 °C. The EIS and LPR techniques were used to estimate the corrosion rate of steel. Figure 1 shows the corrosion rate for a period of 5 h at an exposure test of day 3, which was found to be in the range of 4.10^−4^ and 8.10^−3^ mpy. It was evident from the results that slight variations between the assemblies’ contact surfaces triggered considerable variation in the measured corrosion rate. The problem was tackled by replacing the wire with carbon steel plate (amn X-65) during the developments of high-pressure corrosion probes. Additionally, the short response time was decreased by substituting the 0.0022 cm thick Nafion^®^ HP membrane in place of the 0.0178 cm thick Nafion membrane.

Shitanda et al. [69] developed an electrochemical sensor to study the circuit board’s corrosion in the atmosphere. The sensor element based on a pattern of Ag wiring’s single comb-like structure was fabricated using screen-printing technique. The EIS was used to monitor the corrosion and the impedance data was obtained in wet and dry conditions. The impedance reduced instantly on the generation of water films and then increased slowly in wet conditions. Furthermore, a capacitive loop also emerged, which became large with the growth of water film. The sensor was also investigated for corrosion in a dry sulfur gas, which showed that the sensor’s resistance was comparative to the sulfur atmosphere’s exposure period.

Nyrkova et al. [70] employed an R_p_ and multi-electrode sensors approach to determine the corrosion in metallic structures operating in the atmospheric conditions. The aim of the study was to monitor and predict the protection of metal structures against atmospheric corrosion in humid air (humidity of 100%) and at different temperatures (24, 40, 50 and 70 °C). The obtained results revealed that the accuracy and sensitivity of the corrosion rate measurements in humid atmospheres could be increased by manipulating the thickness of the insulating interlayer, the number of electrodes and their width.

**Figure 2:**
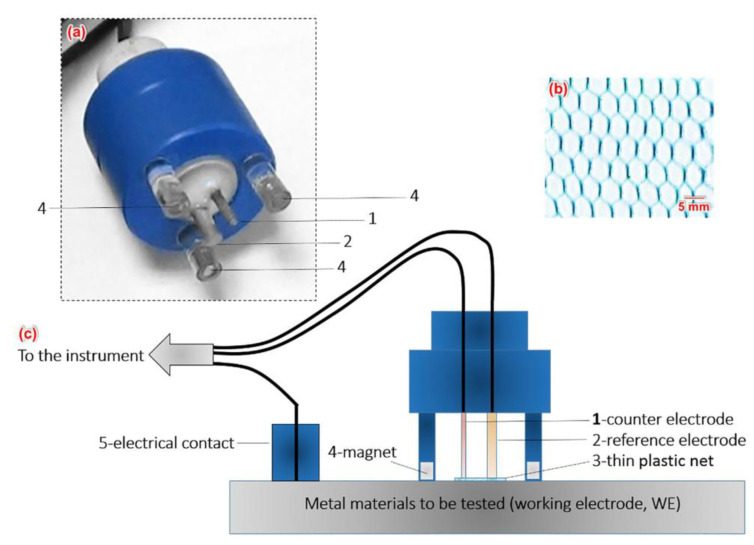
A new electrochemical sensor for atmospheric corrosion monitoring (**a**) a photo of the sensor (**b**) the thin plastic net used as the electrical insulating material (**c**) a sketch map of the sensor during corrosion monitoring. Reproduced with permission from ref. [71]. Copyright 2017 Elsevier.

Xia et al. [71] developed a novel electrochemical sensor for monitoring online corrosion and corresponding corrosion forms of metallic structures in atmospheric conditions. The electrochemical sensor was fabricated by combining it with a thin insulating net, as shown in Figure 2. The EN technique was used to monitor the corrosion rate.

Ma et al. [72] also used EN to analyze the corrosion of haze under the atmosphere of urban Tianjin by using two electrochemical corrosion probes made by Q235B and T91 steels. Experimental results indicated that relative humidity, temperature and concentrations of contaminants could affect the corrosion of Q235B and T91. The corrosion rate in a haze for Q235B and T91 was faster in humid condition than in the dry environment and it was also found that the T91 probe showed better corrosion resistance results than the Q235B probe. They suggested that corrosivity of haze plays a vital role in promoting the corrosion behavior of both Q235B and T91 steel and based on the corrosion behavior, it was concluded that Q235B is suitable for the short time and high-precision detection of the haze corrosivity while T91 probe is suitable for long-term sensing of the corrosivity of haze.

Aiba et al. [73] developed a printed type corrosion sensor that could monitor change in response depending upon the presence or absence of dew condition. The impedance was measured by dropping the NaCl solution on the sensor to simulate dew condensation. The electrochemical impedance measurement showed that the diameter of the impedance spectrum changed in proportion to water film growth. The Nyquist plot converged on the real number axis in the dry condition and showed a wet condition’s capacitive semicircle. The diameter of the capacitive semicircle was proportional to the wet area-ratio of the sensor surface.

Several studies have reported to monitor corrosion in downhole applications under CO_2_ environment and high pressure. Wang et al. [74] developed a suitable sensor for measuring the concentration of CO_2_ (corrosive agent) in real-time at storage depth, that is, in high-pressure conditions. The performance of the CO_2_ sensor was tested by initially put the sensor in a tightly closed stainless-steel tank, contained 0.2 M citric acid. Two ISCO pumps were used to add liquid CO_2_ and brine into the tank. A pressure meter was also connected for stabilizing the tank pressure during the experiment. NaHCO_3_ solution was added to increase the CO_2_ concentration in the solution. In an acidic environment, all carbonates and bicarbonates were transformed into CO_2,_ which increased its concentration in the solution. The additional CO_2_ molecules in the solution diffused through the gas-permeable membrane of the CO_2_ sensor and the corresponding changes were recorded. The sensor element consisted of Ir/IrO_x_ and Ag/AgCl electrodes placed parallel in a porous stainless-steel cup with two connected wires (gold and silver wire), bicarbonate electrolyte solution and a gas-permeable membrane. The pH of the sensor’s internal electrolyte solution was changed with a change in CO_2_ concentration, which was measured in real-time. The sensor displayed a very good response at different CO_2_ concentrations and pressures (1000, 2000 and 3000 psi). A linear relationship between the sensor response potential and the logarithm of the CO_2_ concentration was achieved.

Kahyarian et al. [75] examined the dissolution reaction of iron in aqueous solutions containing CO_2_. The kinetics of the iron dissolution reaction was studied by polarization curves acquired at pH 5 and pH 4, a high corrosion rate was observed in CO_2_ saturated brines than strong acid solutions. The resistance of solution was recorded by EIS measurements at 0 mV DC potential, 5 mV AC potential, and100 kHz to 0.2 Hz frequency range for 15 min after polarization measurements. It was observed that the Tafel slope was decreased in the presence of CO_2_ at low pressure and dissolution range. A Tafel slope of ~120 mV was recorded in the pre-passivation range and significantly depended on CO_2_. They found that the presence of CO_2_ in aqueous solutions significantly affected the iron dissolution reaction at low pressures (1 bar).

In another study, Wright et al. [76] investigated the effect of concentration of HCO_3_^−^ on CO_2_ corrosion of X65 carbon steel (commonly used for transmission pipeline handling oil and natural gas) in 3.5 wt.% NaCl brine solution. The electrode system using a rotating disk working electrode of X65 carbon steel, platinum wire counter electrode, Ag/AgCl reference electrode were used for monitoring the corrosion. The LPR, linear swift voltammetry (LSV) and EIS electrochemical techniques are used for in-situ measurement of the corrosion. The solutions for experiment were prepared with deionized water and N_2_ or CO_2_ was passed through the solution for 90 min to test the effect of N_2_ and CO_2_ environments on corrosion. The LSV measurement showed one order higher anodic and cathodic current densities in CO_2_ saturated solution as compared to N_2_ saturated solution. The LPR measurement showed that the corrosion of the steel has been significantly accelerated in the presence of CO_2_ and could be decreased by adjusting the pH with NaHCO_3_.

Zhao et al. [77] investigated the effect of temperature on X100 steel under dynamic and static conditions in simulated oilfield brines containing saturated CO_2_. The static corrosion and dynamic tests were performed in a thermostatic water bath dynamic corrosion system, respectively. The corrosion of the X100 steel was assessed by employing PDP EIS techniques. The dynamic tests showed 8 times higher corrosion current as compared to that of the static tests at 60 °C with FeCO_3_ and Fe_3_C as corrosion products.

**Figure 3:**
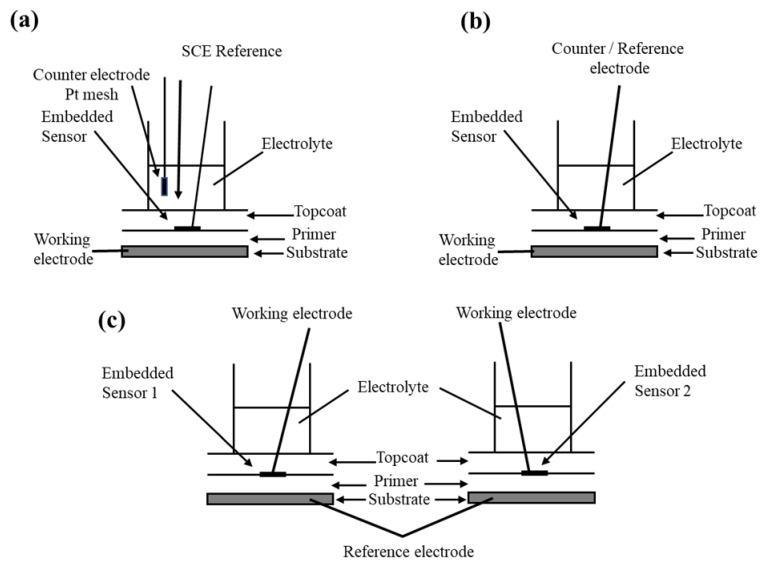
Schematics of the configuration for EIS measurement with (**a**) the 3-electrodes system, (**b**) the 2-electrodes system and (**c**) an EN measurement coupled with the sensors on two panels. Reproduced with permission from ref. [78]. Copyright 2009 Elsevier.

Bierwagen et al. [78] reported electrochemically ac–dc–ac accelerated test method using embedded electrodes in corrosion sensing for aircraft and vehicular structures. EN measurement and EIS methods were applied to monitor the changes in organic coatings. These coatings are comprised of an epoxy primer and a polyurethane topcoat and prepared to deliver different physical characteristics. The measurements were recorded from the changes related to a standard aircraft and an army vehicle coating by the embedded sensors. The coatings were immersed in a 3.5 wt.% NaCl medium and a special method of ac-dc-ac testing was applied to boost the degradation of these coatings. They demonstrated the efficiency of the ac–dc–ac method to step up the degradation of an organic coating with no clear changes in the normal process of degradation. During the experimental process, the samples were exposed to the ac–dc–ac testing for an initial period of 20 days; in this process, the dc signals were kept off to let the samples become equilibrated with the dipping electrolyte. After 20 days, the immersed samples were examined each day by EN and EIS and applied a dc potential cycle for a specific experimental time. The samples could revert to equilibrium for overnight after the dc potential was brought down. During the ac–dc–ac testing of the coatings, −2 and −4 V dc potentials were used. For the first 70 cycles, −2 V potential was used for an application time of 3.5 h while and −4 V potential was used for the last 20 cycles with an application time of 6 h. The schematics of the experimental setup for EN and EIS measurements are shown in Figure 3.

The effect of temperature and flow velocity on corrosion of UNS G10200 steel in aerated brines have been reported by Klapper et al. [79]. The EIS, PDP and LPR techniques were used to study corrosion kinetics of the rotating cylinder electrode (RCE). The corrosion kinetics of the system was enhanced by increasing the flow rate and temperature. Electrochemical parameters and corrosion rates of UNS G10200 steel in aerated 3 wt.% NaCl solution at different temperatures and flow velocities are tabulated in Table 1.

Zheng et al. [80] used a self-made sensor using Zn wire and graphite as reference and counter electrode to develop electrochemical methods OCP, EN, EIS, galvanostatic step and potentiostatic step to infield detect the size of defects in organic coatings exposed to the atmosphere. They demonstrated that, except for corrosion potential, the other four techniques could be used to determine the size of the defect in organic coatings by using R_p_ or R_ct_.

An et al. [81] investigated the corrosion behavior of ZnO nanosheets (NS) (grown on a brass substrate) in different concentrations of chloride solutions. The surfaces were characterized to study the factors affecting the anti-corrosion abilities of the NS. It was concluded from the study that a relatively low concentration (1 wt.%) of NaCl, NSs displays strong passivation on the ZnO but a higher concentration of chloride ions leads to the transformation from ZnO to ZnCl_2_. Moreover, a high concentration of NaCl (3 wt.%) undermines the passivation performance and results in the ZnO NS’s complete dissolution.

Zhang et al. [82] developed an array electrode comprising zinc and mild steel wire sensors to study the galvanic corrosion behavior of a zinc/steel couple immersed in seawater. The electrochemical measurements revealed that there was inhomogeneity in potential and current density in the zinc-steel alloy during galvanic corrosion which was ascribed to the variation in distance between the zinc and mild steel wire sensors over the electrode array as well as their surface electrochemical status. They concluded from the study that the electrode array is a useful technique to examine the electrochemical inhomogeneity of metals/alloys during galvanic corrosion.

The copper alloys are widely used as heat exchanger and condenser tubing materials in power plants due to their excellent thermal and electrical conductivity, mechanical ductility and corrosion resistance. Maria L. Carvalho et al. [83] reported the behavior of online monitoring of corrosion for Al brass and CuNi 70:30 alloys subjected to seawater and corresponding off-line microbiological investigations. LPR technique equipped with electrochemical sensors precisely set for industrial application was used to estimate the rate of corrosion formation. A specific monitoring setup was used in a cooling circuit passing through a marine power plant situated on the Tyrrhenian Italian coast to monitor the corrosion LPR technique. The cooling systems of seawater provide a positive atmosphere for the evolution of micro-organisms that start developing biofilms. Such biofilms cause fouling and adversely influence the performance of equipment and stimulate metal corrosion. Therefore, a specific method is needed to monitor and control these problems through a proper anticorrosive treatment such as online monitoring through the LPR technique. This is a typical example of microbial corrosion detection and interestingly measured by electrochemical methods in the marine atmosphere.

Nie et al. [84] used boron doped diamond (BDD) electrode monitoring corrosion of copper alloys within localized corrosion micro-environments. They studied the electrochemical properties of copper ions on the surface of BDD towards in 0.6 M NaCl aqueous solution. It was observed that in the chloride-containing electrolyte solution, the electrochemical processes for copper ions takes place in two successive single electron transfer steps, as evident from the two well defined separate peaks in a cyclic voltammogram. As compared to perchlorate and sulphate ions, the chloride ions were found to exhibit a stabilizing effect via the formation of CuCl_2_. The pulse voltammetry measurement showed an outstanding relationship between copper ions and the peak current without any significant interference from the commonly present ions. Therefore, it was concluded that BDD could be a promising tool for monitoring corrosion of copper alloys in the marine environment.

Wu et al. [85] used multielectrode array sensors (MAS) to monitor the individual effect of two corrosion inhibitors and single coating on metallic corrosion upon exposure to cyclic deicer condition. The 1008 carbon steel, 304 stainless steel and 1100 aluminum were used to fabricate MAS and the corrosion rate was determined by measuring galvanic current for the MAS probe in the two tested environments, where 3.0% and 2.3% of MgCl_2_ and NaCl solutions were utilized as media, respectively. The corrosion rate and mass loss measurement indicated cyclic exposure of the coated metallic substrate to MgCl_2_ led to pronounced deterioration than NaCl and the finding was validated by EIS and morphological characterization.

A detailed summary of the studies pertaining to the development of corrosion sensor using various working electrodes, interesting electrochemical techniques for monitoring corrosion in natural gas transmission pipeline, atmosphere, CO_2_ and marine environment is given in Table 2.

### 4.2. Corrosion Sensor for Monitoring Corrosion in A Hot Environment

The high-temperature corrosion is more severe when equipment is exposed to varying oxidizing and reducing conditions such as those encountered in boilers operating at high temperature and pressure. To address this type of issue, Covino et al. [86] fabricated LPR-based electrochemical corrosion rate (ECR) probes using mild carbon steel (CS), 304L stainless steel (SS) and 316L (SS) electrode sensors and covered with ash.

The ECR sensors were exposed to a mixed gas environment in the temperature range of 450–800 °C. Mass loss coupons made up of the same material were also employed along with ECR in the same environment to compare corrosion rates obtained from both sources. It was concluded from their experiment that ECR corrosion rates for SS316L and SS304L were similar to those obtained from mass loss coupons, whereas CS showed a disparity in corrosion rates. A comparison of data from EN with LPR demonstrated that the LPR technique could measure the corrosion rate more accurately than the EN technique.

Covino et al. [87] designed electrochemical corrosion rate probes using low carbon steel to determine the operating characteristics in waste-to-energy (WTE) environment. The ash-covered ECR probes along with mass loss coupons were exposed to an environment of were N_2_/O_2_/CO_2_ and water vapor and a temperature range from 200 °C to 700 °C. Three different electrochemical techniques LPR, EN and HDA were applied to study the corrosion rate. The results obtained from ECR probes and mass loss coupons were compared. It was demonstrated that there was a significant difference between the electrochemical corrosion rates and mass loss. Moreover, the LPR based electrochemical corrosion rates of mild steel are a function of the environmental process, temperature and time.

Chiang et al. [88] reported diamond-like carbon (DLC)-coated electrodes for online corrosion sensing under high temperature and pressure. The coupled multi-electrode array sensors (CMAS) probes were developed by multilayer DLC coating on corrosion-resistant Alloy 22 (Ni- 22Cr-13Mo-3Fe-3W) [UNS N06022]) wires and titanium Grade 7 (Ti-0.2Pd [UNS R52400]) electrodes and tested under high pressure and temperature conditions in basic solution (pH 10). They found that DLC films on probes significantly reduced or possibly eliminated crevice corrosion and the effective corrosion monitoring capability of the new electrochemical sensor at high temperatures was attributed to the pinhole-free microstructure, high corrosion-resistance properties of the diamond-like carbon films and high electrical impedance.

The combustion of biomass with coal in pulverized fuel plants has the possibility to produce substantial corrosion problems due to the release of aggressive species from biomass. The deposition of these species in the hot gas path leads to the breakdown of protective oxide films and produces corrosion. Mabbutt et al. [89] fabricated three identical sensors from the same materials as a heat exchanger to investigate the sensitivity of the sensors and monitoring technique, EN in this case considered, as a function of variation in deposition and temperature and different concentrations of SO_X_ in the simulated combustion gas environment. The noise data were achieved by measuring the instantaneous current between the working electrodes at 1 s time intervals. The potential difference is subsequently simultaneously between this couple of working electrodes and a third pseudo reference electrode. The findings indicate that appropriate calculation of resistance values gives a real indication of corrosion rates where general, uniform corrosion occurs for the tested alloys.

Electrochemical based sensors containing diamond-like carbon coated (DLC-coated) electrodes are efficient tools for online real-time monitoring corrosion at high temperatures. Chiang et al. [90] presented a coating deposition method to fabricate sensor electrodes and coupled multi-electrode array sensors (CMAS) for high pressure and high-temperature applications. The sensing probes were prepared using uncoated and DLC coated Titanium Grade 7 and Alloy 22 and electrodes. High pressure and high-temperature conditions were used to test the Alloy 22 probes in a pH 10 basic solution, while the Titanium Grade 7 probes were analyzed in a saturated H_2_S-CO_2_ NaCl solution.

Hot corrosion is a type of oxidation that affects alloys subjected to combustion gases at high temperatures, including small quantities of specific impurities. Na_2_SO_4_ is frequently observed to be the major salt in a hot corrosion system due to its high thermodynamic stability and is an oxidizing gas. Aung and Liu [91] demonstrated Ni-based super alloy (inconel alloy 740) as an electrochemical sensor in a high-temperature system with a consistent reference electrode for corrosion degradation. The sensor’s hot corrosion performance was analyzed using artificial coal ash and an artificial vent gas composed of sulfur dioxide using the high-temperature electrochemical sensor. PDP, EIS and EN measurements were applied to report the sensor’s corrosion performance in a synthetic coal ash environment. No noticeable defects in the outer layer of the electrode was observed after constant analysis for 10 days. The experimental results suggested that the high-temperature corrosion sensor can be used to detect the hot corrosion rate through various electrochemical techniques.

**Figure 4:**
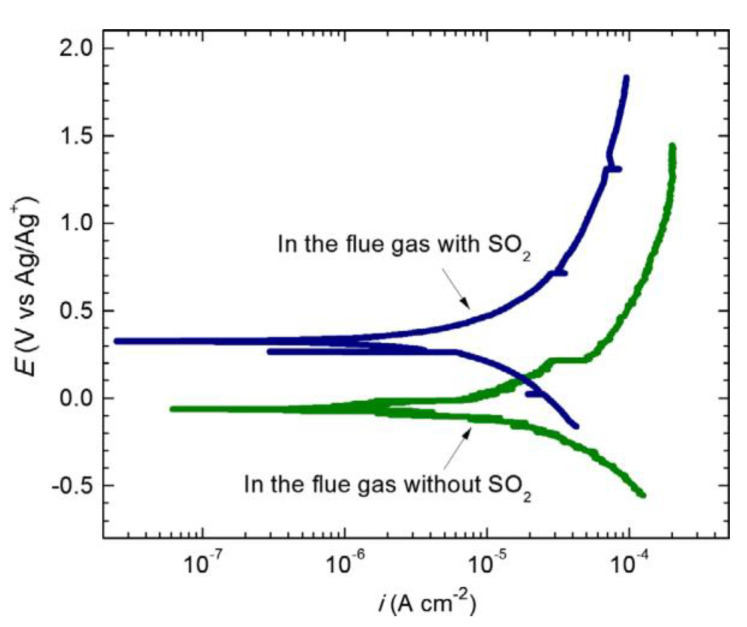
Comparison between Potentiodynamic Polarization (PDP) curves at different flue gas environments. Reproduced with permission from ref. [92]. Copyright 2013 Elsevier.

In another study, Aung and Liu [92] again developed a high-temperature electrochemical sensor for monitoring the effect of SO_2_ in flue gas on coal ash hot corrosion of Inconel 740 alloy. They developed four electrode sensors with two working electrodes, one reference electrode (Ag/Ag^+^ electrode, which contained 10 mol% Ag_2_SO_4_ in the 90 mol% Na_2_SO_4_ molten salt) Pt wire counter electrode. They made the working electrodes using Inconel 740 alloy. The electrochemical PDP, EN and EIS techniques were used to monitor the hot corrosion. Figure 4 presents the PDP curves obtained for the flue gas with and without SO_2_. The corrosion potential without SO_2_ (−68.35 mV vs. Ag/Ag^+^) was lower than with SO_2_ (327.75 mV vs. Ag/Ag^+^), while the corrosion rate and anodic current density were higher with SO_2_ than without SO_2_. Upon analyzing the PDP curves, they concluded that the corrosion rate of Inconel 740 alloy in the flue gas was higher with SO_2_ than without SO_2_. The obtained response of PDP test data was consistent with the OCP, EN and traditional weight loss measurement.

Shi et al. [93] developed an EN sensor for monitoring corrosion of 304 nuclear-grade (NG) stainless steel (SS) in the high purity water system at different temperatures (105–293 °C) and pressures (0.6–19 MPa). They made a working electrode using a 304 nuclear-grade (NG) stainless steel (SS) steel and a reference electrode using the Sb rod. They concluded that a general form of corrosion for 304 NG SS under high purity water, at high temperature and high-pressure system that did not change with temperature.

The Corrosion sensors for monitoring corrosion in a hot environment are summarized in Table 3.

### 4.3. Corrosion Sensor for Monitoring Corrosion in Concrete

**Figure 5:**
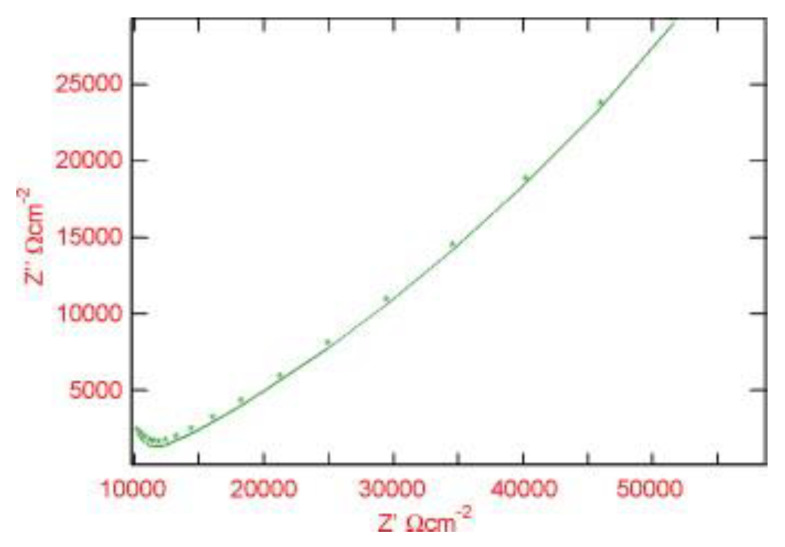
Impedance plot for MnO_2_ sensor embedded in concrete with 0% Cl^−^ and submerged in saturated calcium hydroxide solution. Reproduced with permission from ref. [94]. Copyright 2008 Elsevier.

Corrosion of reinforced concrete structures is a worldwide problem that causes premature degradations, and it can be monitored by embedding a corrosion sensor within the concrete cover. Several studies are available reporting the development of embedded sensors in concrete structures to monitor the corrosion. Muralidharan et al. [94] developed and characterized an embedded MnO_2_ corrosion sensor for monitoring corrosion in concrete structures. The polarizability, stability, impedance and reversibility of the developed sensor were analyzed with respect to some known references. The performance of the MnO_2_ sensor showed a difference of ±5 mV between the reverse and forward scan, which reveals the improved reversibility performance in concrete structures. A half-cell potential of MnO_2_ sensor with reference to saturated calomel electrode and a rebar potential (E_R_) of steel with reference to MnO_2_ sensor was observed for concrete samples for the period of curing. The E_R_ of steel samples with reference to the MnO_2_ sensor are −525 and −315 mV for active and passive rebar circumstances in concrete. Six different sensors have been examined to each test to know the reproducibility of the sensor. Cyclic polarization experiments for the MnO_2_ sensor was performed using a field machine ACM instruments UK in a potential range of −20 to +20 mV with a sweep rate of 1 mV s^−1^. A 3-electrodes system was used which consist of saturated calomel electrode, MnO_2_ sensor implanted in concrete and stainless steel. Cement extract, saturated calcium hydroxide and concrete pore was a test solution for the contact between external reference electrode and the concrete. The experiment was conducted at room temperature and a time span of 10–15 min was provided to each approach for reaching to a steady-state. The AC impedance behavior of the MnO_2_ sensor embedded in concrete was studied and represented in Figure 5. The C_dl_, R_ct_ and half-cell potential (E_1/2_) values obtained for various systems (S0, a system with 0% chloride; S1, a system with 1% chloride; S2, a system with 2% chloride; S3, system with 3% chloride) are tabulated in Table 4. The E_1/2_ for the MnO_2_ sensor in the S0 system was +200, +204 and +205 mV with respect to concrete pore solution (CPS), cement extract (CE) and saturated calomel electrode (SCE), respectively. In a recent report, Colozza et al. reported a paper-based device for monitoring the deterioration in concrete structures. The device was prepared by screen printing the Ag/AgCl on paper and directly applied to monitor real-time corrosion sensing in concrete [95].

Pereira et al. [96] designed an R_p_ sensor for permanent monitoring of the corrosion condition of reinforced steel. The aim of their study was to establish a correlation between the galvanic currents, *I*gal and the corrosion currents, *I*corr, estimated from the R_p_. The sensor was tested at different temperatures in saturated Ca(OH)_2_ aqueous solutions and under a variety of conditions such as ingress of chloride ions, presence or absence of O_2_. They observed a relationship Icorr ≈ 10^9/2^ *I*gal ^6/5^ between the galvanic and the corrosion current densities. The limiting values of the *I*gal, indicative of the state condition of the reinforcing steel for the designed sensor, were established.

Muralidharan et al. for the first-time reported nickel ferrite (NiFe_2_O_4_) as a potential candidate material as an embedded reference sensor in a concrete atmosphere [97]. The NiFe_2_O_4_ sensor properties were tested in concrete situations, such as extract of ordinary portland cement (OPC), synthetic concrete pore solution and saturated calcium hydroxide solution. The electrochemical stability and reliability tests of the sensor were examined in the concrete environments and the half-cell potential was observed to be −300 mV vs. SCE. The impedance and polarization tests of the NiFe_2_O_4_ sensor showed the stability of the synthesized sensor in highly alkaline concrete conditions. The EIS measurements were carried out for the frequency range 30 kHz–0.1 Hz at the OCP of NiFe_2_O_4_ sensor in concrete environments. For most parts at the lower frequencies, EIS measurements showed Warburg impedance. The R_ct_ was calculated from the difference in impedance at high and low frequencies. The C_dl_ was measured from the frequency where the imaginary component -Z’’ was maximum. The calculated impedance parameters for six different sensors (S1 to S6) with the NiFe_2_O_4_ reference sensor in the three alkaline solutions are given in Table 5.

A corrosion sensor was fabricated for monitoring the state of corrosion in a cover mortar [98]. The sensor was analyzed in cement mortar, with and without adding chloride to check the undesirable effects of chloride contaminated conditions on corrosion of concrete structures. ALPR method linked with a reference electrode (embeddable) was used to determine the R_p_ utilizing built-in sensor electrodes. EIS in the frequency range of 1000–50000 Hz was operated using signal amplitude of 10 mV to acquire the resistance of cement mortar (R_s_). The results showed that the R_p_ is related to the chloride substance and R_s_ is directly related mortar without the addition of chloride. The real (Z′) imaginary (Z″) parts of the sensor cell EIS were recorded.

**Figure 6:**
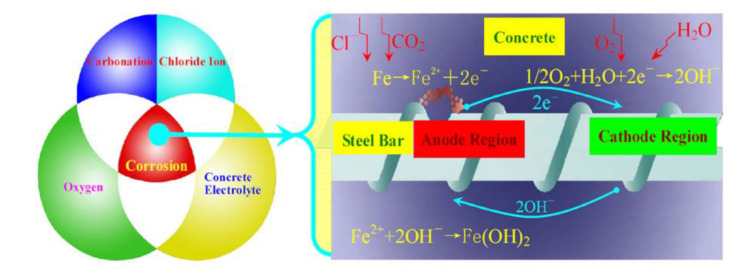
Schematic representation of the reinforcing steel’s corrosion process in concrete. Reproduced with permission from ref. [99]. Copyright 2011 Elsevier.

Guofu Qiao et al. [99] reported that the energy is distributed in the corrosion process as power source and designed an energy harvesting sensor for monitoring the corrosion. Energy harvesting sensors play an important role in uninterrupted monitoring of corrosion. If sensors are only driven by a battery, then the requirements for longer-lifetime, rarely battery recharging/replacing and high-sensing reliability are difficult to be fulfilled. For energy harvesting applications, utilizing atmospheric energy (solar and wind energy) to power up a sensor provides a promising approach to longer time monitoring of corrosion without interruption. A new power source was reported by Guofu Qiao et al., which was extracted from the corrosion process. The energy harvesting sensors for corrosion monitoring were developed by using the new power source. They built a network and applied it to remotely retrieve the corrosion data through active and passive monitoring techniques. The major factors, which produce corrosion in reinforcing concrete (RC) structures and the processes of corrosion processes in the reinforcing steel are demonstrated in Figure 6. A conventional three-electrode electrochemical system was used to monitor the variation in current between the working electrode (WE) and counter electrode (CE) and potential between the WE and the reference electrode (RE). This identifies the micro energy supplied during the corrosion process of the reinforcing steel.

The electrochemical method is one of the popular and reliable methods for corrosion sensing in concrete. However, the success of the corrosion sensing partially depends on the performances of the reference electrode. The liquid reference electrodes, such as the standard hydrogen electrode (SHE), the copper/copper sulfate reference electrode (CSE) and the saturated calomel electrode (SCE) and so forth, are typically used for most laboratory experiments due to their reliable performance. However, due to their fragile nature and the short service life, these reference electrodes are not feasible for field applications. As a result, several embeddable and solid-state REs [98,99,100,101,102,103] and metal-metal oxide (MMO) [104,105] have been developed and tested in RC structures recently. Due to the low survival rate of the above-mentioned solid-state reference electrode, Qiao et al. [106] developed a novel reference electrode based on NiFe_2_O_4_ film for the corrosion monitoring of RC structures. The electrochemical features of the developed reference electrode, including potential stability, the influence of typical ions and temperature response, were investigated and verified.

Xu et al. developed a new corrosion sensor based on electrochemical polarization dynamics for monitoring the corrosion state of concrete cover. They evaluated performance in cement mortar, with dry-wet cycle tests to accelerate the chloride ingress rate. They also observed that the corrosion sensor could efficiently monitor penetration of chloride into concrete with a small influence of the relative humidity in the concrete and concluded from their study that the Ohmic-drop effect can be neglected to makes the examined electrochemical parameters more accurate.

**Figure 7:**
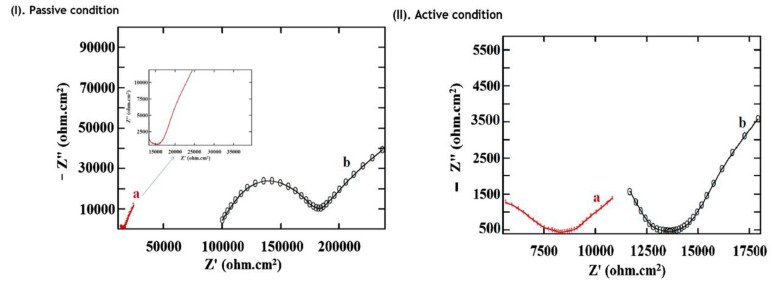
Nyquist plots for steel reinforcement under the active and passive condition in a concrete cube (**a**) embedded sensor and (**b**) surface mounted electrode. Reproduced with permission from ref. [107]. Copyright 2014 Elsevier.

**Figure 8:**
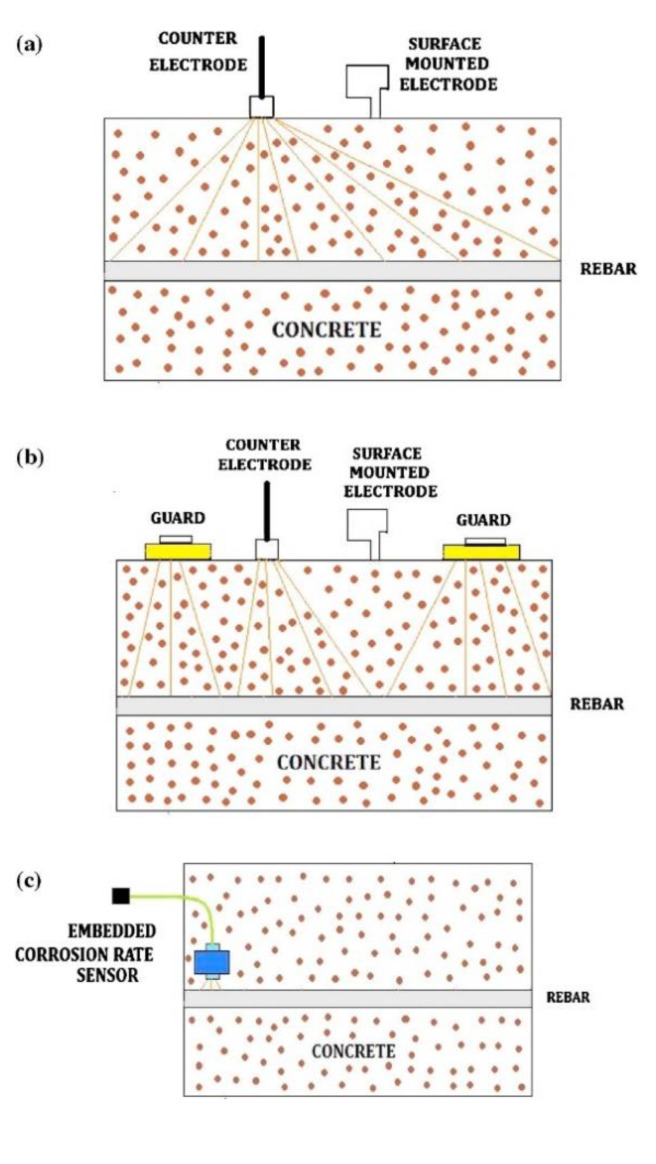
Design of sensor assembly (**a**) surface mounted, (**b**) guard ring and (**c**) embedded corrosion rate monitoring probe sensor. Reproduced with permission from ref. [107]. Copyright 2014 Elsevier.

An embedded MnO_2_ based embedded half-cell potential sensors were developed with monitoring probe and its performance was evaluated in reinforced concrete structures under active and passive conditions of steel reinforcements [107]. The embedded sensor was observed to be stable and indicated precise stability for a period of two years of operation. EIS and PDP were used for the determination of the corrosion rate. The PDP parameters such as E_corr_ and I_corr_ for steel in passive conditions are less than that of active condition; therefore, the embedded sensor apparently differentiated the concrete contaminated by chloride. The EIS findings also showed that the resistance of concrete solution with an embedded sensor is very small but is significant for surface mounted sensors. The I_corr_ values acquired for steel in concrete for embedded sensor was 1.76 × 10^−4^ mA cm^−2^ whereas for surface sensor was 5.10 × 10^−4^ mA cm^−2^. The surface mounted sensor showed 2.89 times higher corrosion rate than the embedded sensor. Figure 7 shows the Nyquist plots for steel embedded in 10 cm × 10 cm × 10 cm concrete cubes with respect to embedded sensor and surface mounted electrode. It was also observed that the resistance of solution for steel is higher in the surface mounted electrode as compared to the embedded sensor. Figure 8 shows the design of sensor assembly (a) surface mounted, (b) guard ring and (c) embedded corrosion rate monitoring probe sensor.

The corrosion rate of carbon steel was determined in four different ways: (i) corrosion sensor based on multiline EIS, (ii) corrosion sensor based on single sine EIS, (iii) weight loss measurements and (iv) corrosion sensor based on LPR attached to an analytical method [108]. After a period of curing for 80 days, the corrosion potential increased to values in the range from 2.1 and 0 V. This increase in the potential of corrosion was assigned to the development of a passive film. The corrosion rate indicated a steep rise after casting to a value of 90 mm/year. This enhancement was ascribed to the growth of the passive film. After 160 days of experiments, the corrosion rate was reduced to 4–5 mm/year.

Sassolini et al. developed an Ag printed electrochemical sensor to detect corrosion level in enforced concrete [109]. The blend of an Ag pseudo-reference electrode with a polymeric gel electrolyte was used for cost-effective, stable and fast potentiometric measurements. These measurements were appropriate for assessing the corrosion of iron bars embedded in concrete models. The potential in concrete based samples comprising chloride (4% *w*/*w*, −0.52 ± 0.01 V) or carbonate (pH 9, −0.35 ± 0.03 V) was observed to be farther negative than in a standard concrete based sample (−0.251 ± 0.003 V). They demonstrated that a printed Ag pseudo-reference electrode combined with KCl offers a reliable and efficient electrochemical measurement for assessing the corrosion of iron bars embedded in concrete-based structures. Table 6 presents a summary of the corrosion sensor for monitoring corrosion in a hot environment with different electrodes, electrochemical techniques, the nature of samples and corrosion behavior.

### 4.4. Corrosion Sensor orf Monitoring Corrosion in Another Environment

An in-situ sensor for corrosion monitoring aids in detecting corrosion in its initial stage much earlier than the visible degradation. This in turn, enhances the development of coatings with quantitative comparison of degradation during accelerated and system service use condition. It also allows maintenance of critical structures based on condition and prevents corrosion failure. Davis et al. reported an in-situ corrosion sensor that can detect corrosion beneath the painting. EIS technique was applied to the sensor, immersed to various accelerated test conditions, for example humidity or salt fog with ambient environments. The EIS measurement enabled the early stage degradation detection of the coated panel before any visible damage allowing a quantifiable comparison between accelerated testing and service degradation conditions [110].

Choi et al. [111] developed chemical sensors based on copper-pipeline steel (Cu-CS) and stainless steel-pipeline steel (SS-CS) to monitor the external and internal corrosion. The damage of the buried pipeline was evaluated by studying the corrosion rates via electrochemical methods under soils of varying resistivity that is, 5000 and 10,000 Ω.cm and synthetic tap water environment. Galvanostatic tests were conducted and the cathodic protection conditions were simulated. Cathodic currents were applied to the pipeline steel for cathodic protection operations and anodic currents were to measure the stray current corrosion. In both cathodic and anodic test potentials of the pipeline steel were recorded. The result indicated that only the Cu-CS probe had a good linear quantitative relationship between the sensor output current and the corrosion rate of pipeline steel in the soil resistivity of 5000 Ω·cm and tap water environments, whereas SS-CS probe showed a better linear correlation than that of the Cu-CS probe in soil resistivity of 10,000 Ω·cm. A correlation based on the ratio of the total charge of the pipeline/Cu-CS probe was determined as 0.39~0.41 in soil environments and 0.76 in synthetic tap water, respectively.

Buchheit et al. [112] described a method to synthesize anion exchange hydrotalcite Al–Zn-decavanadate as a particulate additive to disperse in an epoxy resin for corrosion inhibition of aluminum alloy. The ion exchange characteristic of crystalline hydrotalcite (HT) derived from the release of inhibitor decavanadates and uptake of attacking chloride from electrolyte was utilized for corrosion sensing and inhibition. The decavanadate-chloride exchange reaction’s occurrence indicated the imminent danger of corrosion due to an aggressive electrolyte attack. The anion exchange resulted in a new hydrotalcite phase’s nucleation with different basal plane spacing detected by X-ray diffraction.

**Figure 9:**
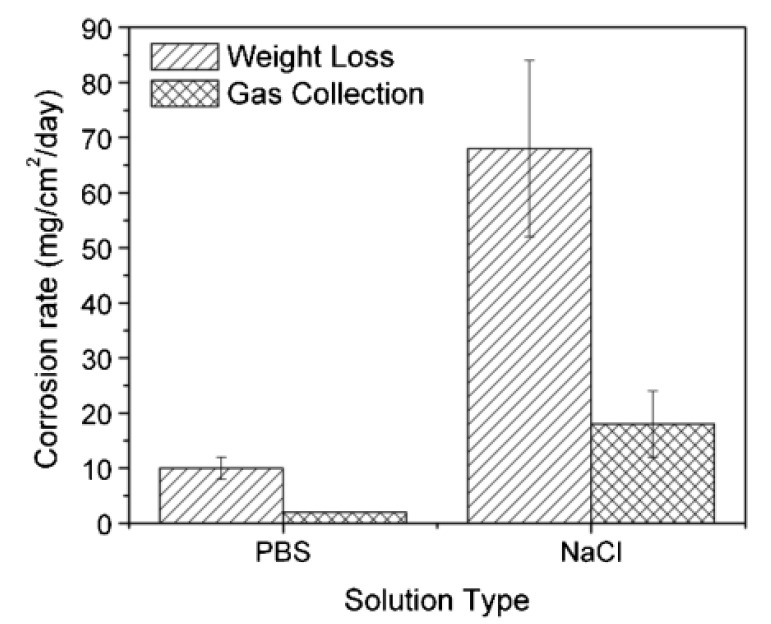
Corrosion rates from gas volume collection and weight loss measurements during the Mg alloy corrosion in 3.5% NaCl and pH 7.4 phosphate-buffered saline. Reproduced with permission from ref. [113]. Copyright 2013 Wiley.

From the last few years, magnesium (Mg) and its composites have focused on the exploitation of bio-degradable metallic materials. A commonly applied technique to analyze the total corrosion rate during the corrosion of Mg and its composite is the measurement of hydrogen (H_2_) gas evolution. Song et al. suggested that the measurement of H_2_ gas evolution is one of the accurate factors determining Mg’s corrosion rate and its composites reliably. Considering the same technique, Kuhlmann et al. [113] have used electrochemical sensing of dissolved H_2_ gas in aqueous solutions to monitor Mg corrosion. They reported a simple H_2_ evolution potentiometric sensor and a Clark-type amperometric microsensor to determine an Mg alloy’s corrosion in aqueous solutions. The sensors performance was monitored in discs of Mg alloys and rare-earth metals (Mg with 0.5% Ga, 0.5% Dy, 2% Nd, 4% Y) immersed in 3.5% NaCl and a pH 7.4 phosphate-buffered saline. The amperometric microsensor revealed that the corrosion was six times greater in NaCl than in phosphate-buffered saline, while the potentiometric sensor indicated a four times higher corrosion. The reported differences in corrosion are due to the effect of the different pH media. The corrosion rate was calculated according to Equation (12) [113]:

(12) $$R=1\times 10^{4}\times \frac{W}{A\times t}$$

where, R is the corrosion rate, W is the weight loss in mg, A is the exposed surface area in cm^2^ and t is the exposure time in days. Another gas collection experiment was used to determine and compare the corrosion rate with weight loss measurements. In this experiment, during the immersion test, a burette and a funnel filled with corrosion solution were placed on Mg alloy’s top. The evolving H_2_ gas displaced the corrosion solution and the collected gas volume in the burette was used to determine the corrosion rate by Equation (13):

(13) $$R=2.279{\;V}_{H_{2}}$$

where, R is the corrosion rate and $V_{H_{2}}$ is the collected volume of H_2_ gas. Figure 9 showed the corrosion rates calculated from weight loss measurements and gas collected volume. Both methods showed the same trend as a higher corrosion rate in 3.5% NaCl than phosphate-buffered saline.

It is important to achieve the relevant information on the state and rate of corrosion inside the instruments to develop effective preventive measures. Elsener et al. [114] developed a non-destructive and small embedded electrochemical sensor for in-situ measurements of corrosion potentials and corrosion rate on several tuning slides various brass instruments. The electrochemical sensor consisted of an Ag/AgCl (pseudo) and a small platinum grid as a reference electrode and counter electrode, respectively. The OCP and R_p_ were measured and a plot of polarization resistance (log R_p_) vs. OCP was constructed from OCP/Rp data obtained from the tuning slides under laboratory established surface conditions.

In another study, Barat et al. [115] developed an electrochemical sensor for corrosion detection on cultural heritage. They optimized the G-PE cell design for in-situ EIS measurements on metallic cultural heritage and to characterize in detail the potential effects of the design of the cell on the EIS spectra. They demonstrated that the cell could be used with a real as well as a pseudo-reference electrode, as desired. In case when a real reference electrode is used, care should be taken to keep it a distance from working electrode surface to prevent pseudo-inductive effects. While in the case of a pseudo-reference electrode, it is desired to place it closer distance to reduce the uncompensated resistance.

Xia et al. [116] identified the corrosion-induced metal release in energy drink by means of electrochemical and inductively coupled plasma mass spectrometer (ICP-MS) analyses. The EIS and EN results showed that coating resistance, R_ct_ and noise resistance decreased with the storage time, which indicated that the corrosion beneath the organic coating induced metal release and a clear and direct relationship was obtained between ICPMS and electrochemical data. They concluded from their study that an electrochemical sensor applied for in-situ measurement could be used for the evaluation of corrosion extent and metal release in beverage cans in a more economical and rapid way.

Wang Ke et al. [117] designed an electrochemical sensor to investigate tinplate cans’ corrosion performance by electrochemical measurements employing EIS and EN techniques. Based on the number of loops and the amplitude of currents in EIS analysis, they concluded that the organic coatings provided protection at an early stage. With an increase in storage time, the coffee permeated to dissolve the tin. When the tin corroded, carbon steel started to corrode, as obvious from the tin and Fe concentration in the medium determined by ICP-MS. The results revealed that the coating resistance, R_ct_ and noise resistance decreased with prolonged storage time.

In another study reported by Wang et al. [118] designed an electrochemical sensor with platinum plating niobium wire as counter electrode and antimony as a reference electrode to evaluate the shelf life of the tinplate cans using OCP, EIS and potentiostatic step techniques and demonstrated that their sensor could be successfully applied to study the shelf life of packaging cans.

Zhang et al. [119] developed an electrochemical sensor for corrosion sensing in the grounding grid without digging out the ground grid or stopping its operation. The corrosion sensing principle was based on the measurement of R_p_. The effect of sensor insertion depth into the soil and hole diameter on the measurement area was studied by measuring the R_p_ of the grounding grid. They obtained the sensor’s optimum diameter as 5 mm by measuring the R_p_ of the grounding grid and concluded that insertion depth has little effect on current confinement at a distance of 12.5 cm.

Nazir et al. [120] demonstrated the effect of temperature, relative humidity and hygroscopic salts contaminants on military vehicles’ atmospheric corrosion using micro-sized LPR sensors. The corrosion rate for operational (uncontrolled environment) and non-operational vehicles were continuously monitored over three years to accumulate the real-time corrosion data to study the structural deterioration and the effect of temperature humidity and hygroscopic salts on corrosion rate.

Corrosion under insulation (CUI) is a costly and dangerous issue affecting the equipment significantly. It is difficult to detect CUI; therefore, a real-time monitoring is an essential part of discovering, managing and avoiding this critical problem. EN is a favorable method that can be employed to pieces under insulation. Caines et al. [121] demonstrated a simple promising EN method that uses electrochemical potential noise (EPN) measurements to predict corrosion time and the system’s mass loss rate. By applying three identical electrodes of the same materials, a basic methodology was introduced using electrochemical potential noise (EPN) to forecast the corrosion rate of a specific electrode and explained its application over short term analysis. 15% NaCl and distilled water solutions were tested and showed a correlation between mass loss and specific electrode potential areas.

**Figure 10:**
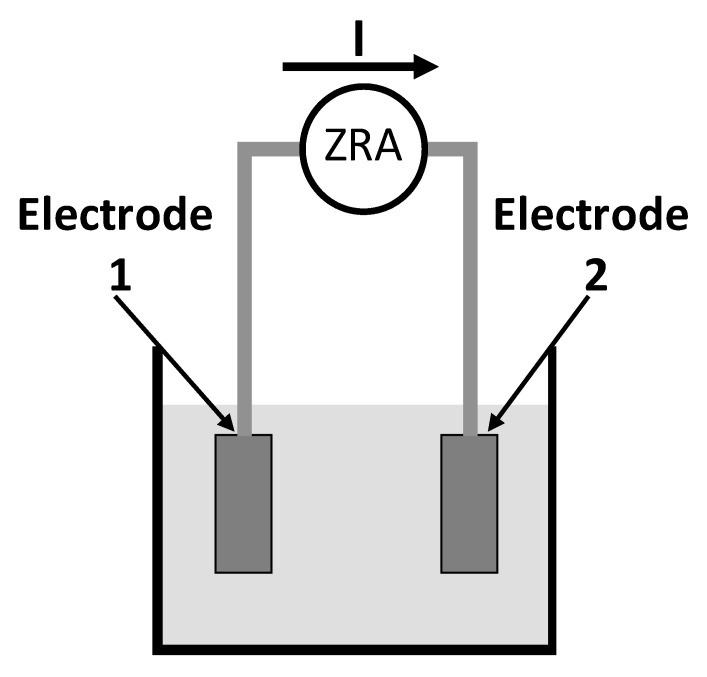
Schematic of the experimental setup for EN measurements. Reproduced with permission from ref. [122]. Copyright 2007 Wiley.

G. Schmitt [122] demonstrated the basic principle of a new EN measurement method using the Coulombs counting method as an online monitoring tool for corrosion monitoring in a wide range of applications. It was indicated that corrosion monitoring could be attained with EN sensors as straightforward as temperature monitoring. This technique can investigate the crevice corrosion under real-time situations. This EN method pays attention to the signals of decaying systems and transforms it into scientifically appropriate data. In this approach, two electrodes are subjected to free corrosion conditions in a designated corrosive atmosphere. In order to determine the charges exchanged between the two electrodes, both the electrodes are connected to a zero resistance amperometer (ZRA). The exchange of charges produces current noise signals measured by a simple system, as shown in Figure 10.

Table 7 summarizes the corrosion sensor for monitoring corrosion in other environments with different working electrodes under various electrochemical techniques.

## 5. Existing Corrosion Monitoring Challenges in Surface and Downhole Conditions

Corrosion is a huge problem in the oil and natural gas (O&G) industry. It has a negative impact on infrastructures utilized for exploration, production, processing and transport, which affects costs and safety considerations [10]. Carbon steel is commonly used in the O&G industry for transmission pipes, casing tubing and drill pipes due to its mechanical properties and economic cost. However, it is prone to corrosion in operating conditions. The proper mitigation/maintenance and real-time corrosion monitoring are crucial to maintain the corrosion rates within an acceptable range to ensure that the infrastructures (e.g., pipes) meet the designed service life. There are several parameters, which make difficult and challenging to monitor corrosion under real condition.

Broadly speaking, major challenges are increased temperature in sub-surface, pressure in downhole generated by various external and internal parameters, the concentration of CO_2_, water and H_2_S makes it difficult to monitor real-time corrosion. Though various methods exist for the detection but still these above-mentioned challenges make it difficult to detect/monitor real-time corrosion. Following are major challenges:

### 5.1. Sub-Surface Temperature

The growing demand for oil and gas all over the world has forced the petroleum industry to drill more deeper wells. The well depths of 25,000 ft. (7620 m) and more are now common and even deeper wells are anticipated in the future with increased pressure and temperature. In the high-pressure/high temperature (HPHT) wells, the temperatures and pressures at the bottom of the well exceed 300 to 350 °F (149 to 177 °C) and 10,000 psi (69 MPa), respectively. The conditions exceeding 400 °F (204 °C) and 20,000 psi (138 MPa) at the bottom hole have been labeled variously as Extreme HPHT (xHPHT) and Ultra HPHT. The xHPHT) and Ultra HPHT conditions are challenging for both the materials and material engineers in the petroleum industry for the future [123] as high temperatures will generally lead to higher corrosion rates, given that no corrosion product layer forms [124]. At higher temperature conditions, both the chemical reaction in the bulk solution and the electrochemical reactions at the metal surface gets accelerated giving rise to increased corrosion rates. Moreover, the solution’s viscosity is also decreased at higher temperatures leading to an accelerated mass transfer process. Normally the corrosion rate reaches a maximum with increasing temperature at around 70–90 °C. So, if the protective films are not formed (especially at low pH), the general CO_2_ corrosion rate will increase with temperature. However, at higher pH, with increasing temperature, the kinetics of precipitation accelerates and aids in the protective film formation, which was ascribed to the formation of a protective film of FeCO_3_ corrosion product [125].

Additionally, variation in the temperature of the environment in which the pipeline is situated also affects the corrosion rate by changing its electrical resistance. For example, the resistance of steel may change by 0.4% per °C.

In corrosion monitoring systems measuring electrical resistance, if in case the monitoring element and the reference element have exposed surfaces in different environments such as outside and inside the fluid environment, any variation in fluid temperatures significantly limit the accuracy and sensitivity of the monitoring system if the temperature of the pipeline and external reference system differ. For instance, a minimal difference in temperature of 0.25 °C between the pipeline and reference system will change the resistance ratio of 1000.

In downhole applications, the physical conditions are more stringent with high temperature, high pressure, presence of highly corrosive fluids and gases as well as high produced fluids velocities. In particular, high pressure plays an important role in most mechanisms affecting the well integrity, including corrosion progression on the tubular walls and casings. Mitigation methods such as internal coatings of tubular are limited with current technologies due to the high temperature and pressure conditions downhole. Therefore, installing continuous corrosion monitoring systems in the well is always problematic, especially that most currently available corrosion sensors occupy some space, limiting the flowing area especially in production tubing leading to the need for small sensor platforms to monitor corrosion progression downhole.

### 5.2. Corrosive Chemical Environment

Corrosive environments downhole can be caused by the presence of chlorides, CO_2_, H_2_S and so forth. Pressure, temperature, pH and so forth also affect the corrosivity of downhole components [126]. Chlorides can be extremely corrosive and usually drives localized corrosion like pitting and crevice corrosion. This is increased at higher temperatures as with other methods of corrosion. Corrosion due to CO_2_ and H_2_S present in condensed water phases have been a matter of concern for the engineers and scientists handling operations, from extraction through processing. Most commonly, corrosion starts in the presence of water (aqueous media). When CO_2_ is present, it reacts with water forming carbonic acid, which then corrodes the iron, forming an iron carbonate scale. In some instances, this scale tends to form a protective covering over the iron, preventing further corrosion. In regions of high flow rates and turbulence, these scale coverings can breakdown, leading to extremely high corrosion rates and so these scale coverings cannot be relied on to protect against corrosion. The major effects of sweet corrosion are that it increases corrosion rate due to low solution pH caused by dissolved CO_2_. The presence of hydrogen sulfide is mainly dangerous to equipment when moisture is present. Sour corrosion, where H_2_S is present at low concentrations, has generally been shown to reduce corrosion rates due to quick formation of protective iron sulfide film as passivation. The presence of H_2_S at levels exceeding partial pressures of 5 psi makes the artificial lift equipment susceptible to sulfide stress corrosion cracking. However, while the reduced material loss may be welcome, H_2_S content’s tradeoff increases the susceptibility for sulfide stress cracking (SSC). With drastically different failure modes being possible, mixed systems’ behavior becomes important for the proper design of corrosion prevention and mitigation [127]. Further, in some instances the presence of polysulphides, organic acids and elemental Sulphur can create special problems.

One of the additional affecting parameters in corrosion progression in downhole environments is the presence of water or brine. Both in oil and gas wells at a certain stage of the production, water is produced at different percentages depending on several factors. Most of the time produced water has very high salinities in the order to hundreds of thousands of ppm of total dissolved salts, which make these fluids very corrosive at downhole conditions. In flowing lines as well as treatment equipment, the produced water also affects the integrity of the equipment due to potential high corrosion rates that will lead to equipment failures in particular. Therefore, it is primordial to monitor the amount of produced water first downhole in order to anticipate any potential corrosion consequences by installing downhole sensors and gauges that can measure water salinity and water contents in addition to other corrosion measurements.

### 5.3. Distance

In the petroleum industry, oil and natural gas are transported to long distances by pipelines covering thousands of miles in length. The failure analysis of these pipelines is important for the industry. Therefore, it is of utmost importance to locate the corrosion phenomenon along the long-distance pipelines for real-time corrosion monitoring [10]. Due to their long-distance, it has been challenging to monitor the internal corrosion, particularly inside the pipelines, as these areas are not accessible to during inspection and maintenance. Moreover, the location of corrosion is not fixed and can occur at random locations in the interior or exterior parts of the pipelines.

In downhole applications, corrosion monitoring causes additional problems in terms of sensor communications, especially in a gas well that are deep in the sub-terrain. Real-time corrosion monitoring downhole would need communication through electrical lines; ideally, however, this is not always possible depending on the well completion. In deep wells, it is preferable that communication is performed wirelessly. Therefore, the corrosion monitoring platform’s architecture and design must overcome the challenge of the well environment and well monitoring distance.

### 5.4. Data Acquisition

The telemetry system is used to transmit the sensor data to the surface and the control signals from the surface to the respective control downhole. The drawbacks associated with active, embeddable sensors make them undesirable for use in a wellbore environment. As an example, nanowatt size electronic moisture sensors are available, but it has intrinsic limitations when embedded within the cement. The sensor’s electronic elements are prone to damage in the highly alkaline conditions and are sensitive to electromagnetic noise. Additionally, to activate the sensor and transmit data, power must be provided from an internal battery, increasing the sensor size and decreasing sensor life [128].

As previously mentioned, in downhole corrosion monitoring, the main handicap is communicating the data from the sensor to the surface. In retrievable systems, such as the intervention application via wireline, for instance, the electrical line transmits the sensor data to the surface in real-time. However, if the sensor is permanently installed and is the preferable case in corrosion monitoring, data communication becomes crucial. The insertion of a cable might complicate the well completion. However, wireless communication is an attractive solution which has current limitation regarding the length of transmission. On the other hand, memory gauges have also been used for several monitoring aspects. Corrosion could be one of these. In this case, frequent intervention and data collection are needed that complicate the monitoring operation and increase operational costs.

## 6. Outlook and Conclusions

Corrosion poses one of the unique challenges for drilling technology and the health of the drilling equipment and pipe-line integrity on the surface and under the downhole and offshore conditions. Several reasons can be attributed to the corrosion, such as geographical distances these tools and pipelines cover, their burial underneath the soil/sea in different depths, materials age and nature of the product which flows through pipelines. At present various methods are explored to monitor corrosion in downhole/offshore/surface. However, there is an urgent need to design effective and precise corrosion detectors that can save huge economic losses due to unnatural shutdown, pipeline health and many other industrial installations. Thus, a corrosion sensor’s development would involve a search for innovative techniques that are amenable to develop probe to detect corrosion rate and scale formations more precisely on surface and sub-surfaces. To address this challenge, it is necessary to understand various types of corrosions occurring in surface and downhole conditions. In this work, we have comprehensively reviewed various types of corrosion and their chemical environment. Understanding the mechanism and causative factors of corrosion is helpful to develop more reliable and promising sensors for corrosion monitoring. In this particular review, we presented recent progress on electrochemical methods to detect corrosion. These methods are low cost, reliable and simple in their design compared to other various systems presently employed to monitor corrosion in different environments on the surface and sub-surface conditions. Furthermore, various intriguing challenges in corrosion monitoring, particularly in downhole conditions, are also highlighted to understand corrosion and identify and solve these problems. For the inclusive application of electrochemical corrosion sensors, there are needs for impending advances related to instrumentation, electrochemical techniques such as PDP, LPR, ER, data acquisition, surface wireless communication systems and exclusive data mining.

1. Development of effective and accurate corrosion sensor technology needs complete information in a downhole/surface/offshore environment, which causes various types of corrosions that can be retrieved by proper data, carry out modeling and so forth.
2. Reliable and miniatured electrochemical sensors can be more effective in detecting the chemical environment, which results in corrosion, particularly in downhole and offshore conditions.
3. Advanced wireless data acquisition tools can be integrated with electrochemical sensors to accomplish proper data collection, communication and alarm.
4. More research on data mining and converting into real information related to corrosion under different environments.

## Figures and Tables

**Table 1:** Electrochemical parameters and corrosion rates of UNS G10200 steel in aerated 3 wt.% NaCl solution at different temperatures and flow velocities. Reproduced with permission from ref. [79]. Copyright 2008 Elsevier.

*T* (°C)	w (rpm)	Rp_DC_ (k Ωcm^2^)	Rp_AC_ (k Ωcm^2^)	β_C_ (mV)	β_A_ (mV)	B (mV)	i_corr_ (μA/cm^2^)
25	0	0.9141	0.8123	24.30	43.98	6.81	7.44
	500	0.2127	0.2085	54.49	146.79	17.28	81.23
	1250	0.1241	0.1245	54.12	123.35	163.35	131.79
	2000	0.1179	0.1057	56.76	111.54	16.36	138.72
35	0	0.6502	0.6315	24.38	41.83	6.70	10.30
	500	0.1388	0.1326	47.60	185.64	16.47	118.67
	1250	0.0759	0.0759	53.28	98.61	15.04	198.15
	2000	0.0691	0.0706	58.62	141.11	18.01	260.59
45	0	0.6166	0.5921	24.20	47.68	6.98	11.32
	500	0.1143	0.1257	47.83	160.91	16.03	140.25
	1250	0.0712	0.0715	54.01	127.90	16.51	231.89
	2000	0.0618	0.0575	52.96	153.04	17.11	276.80

**Table 2:** Corrosion monitoring in natural gas transmission pipeline, atmosphere, CO_2_ and marine environment.

Working Electrode	Electrochemical Techniques	Sample	Corrosion Sensor/Behavior/Casing Agents/Others	Publication Year	Ref.
A106 pipeline steel in the form of flange probes	EN	Natural gas transmission pipelines	Corrosion	2004	[65]
A106 pipeline steel	EN, LPR, HDA	Natural gas transmission pipelines	Corrosion sensor	2005	[66]
Carbon steel electrode	LPR, EIS	Carbon steel in Natural Gas Pipelines	Corrosion	2017	[68]
-	EIS	circuit board	atmospheric corrosion monitoring	2009	[69]
Metal material to be tested	R_p_	St.3 steel	Atmospheric corrosion	2012	[70]
Metal material to be tested	EN	atmospheric corrosion of metals	Corrosion	2017	[71]
Q235B and T91 steels	EN	atmospheric corrosivity of Q235B and T91 steels	Sensing the Instant Corrosivity of Haze	2017	[72]
Screen Printed-Type Ag	EIS	Screen printed type Ag	Corrosion monitoring	2018	[73]
Ir/IrO_x_	Potentiometric	CO_2_ detection in downhole (high temperature and high pressure)	CO_2_ sensor	2016	[74]
A disk-shaped API 5L X65 mild steel	Anodic polarization	CO_2_ corrosion of mild steel	Corrosion	2017	[75]
X65 carbon steel	LPR	X65 carbon steel (It is noted that X65 carbon steel is commonly used for transmission pipeline handling oil and natural gas)	Corrosion sensor	2018	[76]
X100 steel	EIS, OCP and PDP	X100 steel in simulated oilfield brines under the static and dynamic conditions	Corrosion behavior	2019	[77]
Substrate of interest	EIS/EN	Aircraft and vehicular structures Protected by organic coatings in 3.5% NaCl solution	corrosion	2008	[78]
UNS G10200 steel	LPR, EIS, PDP	Corrosion of UNS G10200 steel in aerated brines under hydrodynamic conditions	Corrosion	2008	[79]
Galvanized Q235 carbon steel	OCP, EN, EIS and potentiostatic step	Galvanized Q235 carbon steel with a size of 40 × 20 cm covered by a layer of high solid epoxy resin coating with an average thickness of ~500 μm.	Corrosion sensor	2020	[80]
ZnO nanosheets on a brass substrate	PDP and OCP	ZnO nanosheets on brass substrate	Corrosion behavior	2009	[81]
Electrode array composed of zinc and mild steel wire	OCP	Zinc/steel couple immersed in seawater	Electrochemical inhomogeneity of zinc in zinc/steel couple during galvanic corrosion	2010	[82]
-	LPR	Marine corrosion of copper alloys	Corrosion	2014	[83]
BDD	Voltammetry	Copper alloys in chloride background electrolyte	Corrosion monitoring	2016	[84]
1008 carbon steel, 304 stainless steel and 1100 aluminum	Multielectrode array sensors	1008 carbon steel, 304 stainless steel and 1100 aluminum in 2.3% NaCl and 3.0% MgCl_2_	Corrosion sensor	2016	[85]

**Table 3:** Corrosion sensor for monitoring corrosion in hot environment.

Working Electrode	Electrochemical Techniques	Sample	Corrosion Sensor/Behavior/Casing Agents/Others	Publication Year	Ref.
Mild Steel, 304L SS, 316L SS probes	EN, LPR and HDA	The probe in mixed gas environment (O_2_, N_2_, H_2_O and CO_2_) in high temperature (e.g., Inside of boiler/coal combustor)	Corrosion sensor	2004	[86]
Low carbon steel	EN, LPR and HDA	Steel either in an air/H2O or a mixed gas environment identical to a waste to energy (WTE) Environment	High-temperature corrosion	2004	[87]
Diamond-like carbon-coated Alloy 22 (Ni-22Cr-13Mo-3Fe-3W) electrodes	Based on the current measured from the most anodic electrode	DLC-coated Alloy 22 in a solution saturated with NaCl–NaNO3–KNO3	corrosion	2008	[88]
Metal material (either superheater/reheater or waterwall) to be tested	EN	High-temperature corrosion monitoring	corrosion	2009	[89]
Diamond-like carbon-coated Alloy 22 (Ni-22Cr-13Mo-3Fe-3W) and Titanium Grade 7 (Ti-0.2Pd) electrodes	Anodic current (Multielectrode Array Sensors)	online, real-time corrosion monitoring of Diamond-like carbon-coated Alloy 22 (Ni-22Cr-13Mo-3Fe-3W) and Titanium Grade 7 (Ti-0.2Pd) at high temperatures.	corrosion	2010	[90]
Ni-based superalloy (inconel alloy 740)	EN, EIS and PDP	Ni-based superalloy in the presence of a synthetic coal ash and a synthetic flue gas containing sulfur dioxide	corrosion	2012	[91]
Inconel 740 alloy	EN, EIS, PDP	Inconel 740 superalloy	Corrosion sensor	2013	[92]
304NG Stainless Steel	EN	304NG Stainless Steel in high-temperature Water	Corrosion	2014	[93]

**Table 4:** Parameter of AC impedance for MnO_2_ sensor embedded in concrete [94]. Reproduced with permission from ref. [94]. Copyright 2008 Elsevier.

Solution	System	*E*_1/2_ (mV vs. SCE)	*R*_ct_ (×10^4^ Ω cm^2^)	*C*_dl_ (×10^−5^ Fcm^−2^)
SCS	S0	205	2.010	1.620
	S1	206	1.475	1.777
	S2	201	1.424	1.729
	S3	204	1.039	1.588
CPS	S0	200	1.585	1.966
	S1	201	1.432	1.440
	S2	206	1.099	1.078
	S3	206	0.937	1.537
CE	S0	204	1.311	1.189
	S1	208	1.260	1.949
	S2	202	1.248	1.010
	S3	200	0.867	1.355

**Table 5:** Impedance parameters for six different sensors (S1 to S6) with NiFe_2_O_4_ reference sensor in concrete environments. Reproduced with permission from ref. [97]. Copyright 2010 Elsevier.

Sensor Number	OCP (mV vs. SCE)	*R*_ct_ (Ω cm^2^)	*C*_dl_ (Fcm^−2^)
Saturated calcium hydroxide solution			
S1	−293	2.168 × 10^2^	1.111 × 10^−3^
S2	−290	3.718 × 10^2^	1.482 × 10^−3^
S3	−309	4.545 × 10^2^	1.176 × 10^−3^
S4	−307	2.388 × 10^2^	3.256 × 10^−3^
S5	−295	2.736 × 10^2^	2.111 × 10^−3^
S6	−294	2.651 × 10^2^	4.017 × 10^−3^
Average value with standard deviation	−299.5 ± 9.5		
Concrete pore solution			
S1	−291	5.811 × 10^2^	1.566 × 10^−3^
S2	−302	3.391 × 10^2^	1.148 × 10^−3^
S3	−305	6.064 × 10^2^	1.673 × 10^−3^
S4	−309	1.852 × 10^2^	1.850 × 10^−3^
S5	−308	1.846 × 10^2^	1.013 × 10^−3^
S6	−308	1.833 × 10^2^	1.288 × 10^−3^
Average value with standard deviation	−300.0 ± 9.0		
Cement extracts			
S1	−302	2.588 × 10^2^	1.674 × 10^−3^
S2	−306	5.187 × 10^2^	1.144 × 10^−3^
S3	−301	5.422 × 10^2^	1.611 × 10^−3^
S4	−301	2.596 × 10^2^	2.116 × 10^−3^
S5	−302	2.239 × 10^2^	2.980 × 10^−3^
S6	−303	2.069 × 10^2^	1.774 × 10^−3^
Average value with standard deviation	−303.5 ± 2.0		

**Table 6:** Corrosion sensor for monitoring corrosion in concrete.

Working Electrode	Electrochemical Techniques	Sample	Corrosion Sensor/Behavior/Casing Agents/Others	Publication Year	Ref.
Steel	PDP	Corrosion monitoring in concrete structures	Corrosion	2008	[94]
Metallic bar	Potentiometric measurements	Reinforced concrete artefacts	Corrosion	2020	[95]
Carbon steel	Galvanic and potentiostatic pulse method	Concrete reinforcing steel in saturated Ca(OH)_2_ aqueous solutions	Corrosion sensor	2009	[96]
NiFe_2_O_4_ reference electrodes as the working electrode	PDP, EIS	steel in concrete environments	Corrosion	2010	[97]
Steel	LPR, EIS	Chloride-Contaminated Cement Mortar	Corrosion	2010	[98]
Q235 steel	Active monitoring techniques (AMTs) and passive monitoring techniques. AMT covers EIS, HA, transient galvanostatic/potentiostatic decay, potential dynamic scan, LPR and coulostatic method	Reinforcing concrete structures	Remote corrosion monitoring	2011	[99]
Solid-state reference electrode containing NiFe_2_O_4_ film	Polarization, EIS	RC structure	Corrosion sensor	2012	[106]
Steel	PDP, EIS	Corrosion monitoring of steel in concrete structures	Corrosion	2014	[107]
A carbon steel plate	LPR, ESI	carbon steel overpack exposed to super container concrete buffer	Corrosion	2014	[108]
Iron-reinforced concrete specimens	Potentiometric measurements	Iron reinforced concrete	Corrosion	2019	[109]

**Table 7:** Corrosion sensor for monitoring corrosion in another environment.

Working Electrode	Electrochemical Techniques	Sample	Corrosion Sensor/Behavior/Casing Agents/Others	Publication Year	Ref.
Materials of interest	Based on EIS	Coating materials deterioration and substrate corrosion	Corrosion	2000	[110]
Carbon steel	Galvanic	Steel Pipelines	Corrosion sensor	2006	[111]
Aluminum alloy substrate.	EIS	Aluminum alloy substrate	Corrosion Sensor	2003	[112]
Pt disc electrode	Potentiometric	Magnesium Alloy Corrosion in Aqueous Solutions	Magnesium alloy corrosion in aqueous solutions	2013	[113]
Tuning slides of different brass instruments	OCP and LPR	Historical brass wind instruments	Corrosion Sensor	2016	[114]
Stainless steel and Bronze	EIS	metallic cultural heritage	Corrosion Sensor	2018	[115]
Tinplate cans	EIS and EN	tinplate cans	Corrosion	2012	[116]
Tinplate cans	EIS/EN	tinplate cans containing coffee	Corrosion	2014	[117]
Tinplate cans	OCP, (EIS and potentiostatic step techniques	Four lacquered tinplate cans (65 mm diameter X 90 mm high) provided by the ORG Can making Company (China) were used as the investigated objectives	Corrosion sensor	2019	[118]
Carbon steel	Potential time curve	Grounding grid	Corrosion sensor	2010	[119]
Material of structure	LPR	large complex engineering structures	Electrochemical corrosion failure	2018	[120]
carbon steel electrodes	EN	Carbon steel	corrosion	2017	[121]
Materials of metal (e.g., steel, copper, magnesium, etc.)	EN	metals (e.g., steel, copper, magnesium) in diverse media	corrosion	2007	[122]
